# Editorial: Hemophilia advances: from genetic insights to optimal therapy in 2025

**DOI:** 10.3389/fmed.2026.1910117

**Published:** 2026-07-09

**Authors:** Ciprian Tomuleasa, Laszlo Nemes

**Affiliations:** 1 Department of Hematology, Iuliu Haţieganu University of Medicine and Pharmacy, Cluj-Napoca, Romania; 2 Department of Personalized Medicine and Rare Diseases—Medfuture Institute for Biomedical Research, Iuliu Haţieganu University of Medicine and Pharmacy, Cluj-Napoca, Romania; 3 Department of Hematology, Ion Chiricuta Oncology Institute, Cluj Napoca, Romania; 4 National Hemophilia Centre and Haemostasis Department, Central Hospital of Northern Pest—Military Hospital, Budapest, Hungary

**Keywords:** 2025–2026, editorial, genetics to gene therapy, hemophilia, modern approach

## Introduction

Hemophilia A and B are X-linked bleeding disorders caused by deficiency of coagulation factors VIII and IX, respectively. Despite representing a small fraction of all inherited bleeding disorders, they impose a disproportionate burden of morbidity, reduced quality of life, and healthcare expenditure worldwide. The management landscape has been transformed over recent decades by the introduction of recombinant factor concentrates, extended half-life products, and, most recently, non-factor replacement therapies and gene therapy. Yet, profound inequities persist: as many as 70% of patients globally still lack access to adequate treatment, and fundamental questions remain regarding long-term outcomes, pharmacokinetic optimization across the lifespan, and the clinical management of rare, life-threatening complications.

This Research Topic, *Hemophilia Advances: From Genetic Insights to Optimal Therapy in 2025*, published in *Frontiers in Medicine*, brings together seven contributions spanning the full translational arc of hemophilia research: from bench-to-bedside gene therapy and population pharmacokinetics, through neonatal diagnosis and rare complications, to state-of-the-art cardiac surgery and the genomic landscape of closely related myeloid disorders. Together, these manuscripts encapsulate the complexity of modern hemophilia care and point toward the therapeutic horizon that lies ahead.

## Mapping the therapeutic landscape

The most widely read contribution in this Research Topic is the comprehensive narrative review by Dushimova et al. (Al-Farabi Kazakh National University and collaborating institutions, Kazakhstan), entitled “*Therapeutic advances in hemophilia: from molecular innovation to patient-centered global care*.” Attracting over 5,400 views and six citations since its September 2025 publication, this review provides an authoritative synthesis of hemophilia pathophysiology, genotype-phenotype correlations, and the complete spectrum of current and emerging therapies.

The authors methodically examine factor replacement therapy—still the cornerstone of hemophilia management—alongside its well-known limitations: the necessity for frequent intravenous infusions, high treatment costs, and the risk of inhibitor formation. Against this background, they assess the newer generation of extended half-life (EHL) products and subcutaneous non-factor therapies (principally emicizumab), which have demonstrably improved adherence and lowered annualized bleeding rates. Gene therapy emerges as the most transformative prospect on the horizon, with several AAV-based products now approved or in late-phase trials; however, Dushimova et al. are careful to delineate the unresolved challenges: uncertainty about long-term durability of transgene expression, immunological responses to viral vectors, and the underrepresentation of hemophilia B relative to hemophilia A in translational research pipelines.

Critically, the review foregrounds the issue of global inequity. Despite substantial scientific progress, approximately 70% of patients worldwide cannot access advanced therapies, and the majority of the global hemophilia burden falls in low- and middle-income countries where even basic factor concentrates may be unavailable. This observation frames much of what follows in the Research Topic: technical advances are meaningful only insofar as they can be translated into broadly accessible, patient-centered care.

## Non-viral gene therapy: a safer vector for factor VIII delivery

Addressing the immunological concerns associated with AAV-based approaches, Kao et al. (National Chung Hsing University and collaborating institutions, Taiwan) report the long-term outcomes of a non-viral gene therapy strategy for hemophilia A in their original research article “*Non-viral gene therapy for hemophilia A: long-term outcomes of minicircle FVIII delivery in a mouse model*,” published in *Frontiers in Pharmacology* in April 2026.

The investigators evaluated minicircle DNA constructs—vectors from which the bacterial backbone has been excised, thereby reducing immunogenicity—encoding either wild-type FVIII or a stabilizing point mutant (E1984V–FVIII). Constructs were delivered by hydrodynamic tail-vein injection into FVIII-knockout mice, a well-validated murine hemophilia A model. The study demonstrated that minicircle-based delivery sustained FVIII activity and ameliorated coagulopathy for at least 26 weeks, though a gradual decline in expression was noted in the later phase of follow-up. Importantly, the E1984V mutation was engineered to improve protein stability and activity, potentially compensating for expression attrition over time.

This work contributes an important proof-of-concept for non-viral gene therapy as an alternative to AAV vectors. While hydrodynamic injections are not directly translatable to humans, the minicircle platform is compatible with nanoparticle-based delivery systems that are under active clinical development. The persistence of therapeutic FVIII activity over a 6-month observation window is encouraging, and the absence of an integrating vector mitigates concerns about insertional mutagenesis. Future studies will need to address long-term durability, repeated dosing strategies, and scaling to larger animal models.

## Precision dosing: pharmacokinetic modeling of rIX-FP across the lifespan

Translating therapeutic advances into optimized clinical protocols requires sophisticated pharmacokinetic (PK) modeling, particularly for pediatric patients in whom drug handling may differ substantially from adults. Terasaka and McKeand (CSL Behring K.K., Japan, and CSL Behring, USA) address this challenge in their original research paper “*Optimization of hemophilia B treatment via population PK modeling of rIX-FP, including a 3-week regimen*,” published in *Frontiers in Pediatrics* in December 2025.

Using pooled FIX activity data from 113 previously treated patients aged 1–63 years across five clinical studies, the authors developed a two-compartment population PK model of recombinant factor IX–albumin fusion protein (rIX-FP, albutrepenonacog alfa). The model identified body weight as the dominant covariate influencing clearance and volume of distribution, with weight-normalized clearance being substantially faster in children under 6 years—a finding with direct implications for dosing frequency in young children. Notably, no clinically meaningful ethnic effect was detected between Japanese and non-Japanese patients, supporting the generalizability of the model across populations.

Simulations derived from the model demonstrated that patients aged 12 years and above can maintain median steady-state FIX trough levels above the 5% activity threshold on a 3-week dosing interval—a clinically attractive extended regimen that reduces infusion burden considerably. Younger children, however, require more frequent dosing due to their faster clearance. These findings are immediately actionable in clinical practice and provide a quantitative basis for individualized prophylaxis regimens, embodying the shift toward pharmacokinetic-guided, patient-tailored therapy that is increasingly recognized as the standard of care in hemophilia management.

## Neonatal diagnosis: the earliest presentation of hemophilia A

The clinical challenges of diagnosing hemophilia in the earliest days of life are vividly illustrated by the case report of Peng et al. (Peking University First Hospital and collaborating institutions, China), “*Progressive cephalohematoma in a neonate revealing severe hemophilia A owing to intron 22 inversion*,” published in *Frontiers in Pediatrics* in July 2025.

The authors describe a male neonate admitted on day 8 of life with progressive jaundice, a large and expanding cephalohematoma, and multiple ecchymoses. Laboratory evaluation revealed severe anemia and a markedly prolonged activated partial thromboplastin time. APTT mixing studies demonstrated factor deficiency, and FVIII activity measured below 1%, establishing the diagnosis of severe hemophilia A. Genetic analysis identified the intron 22 inversion in the F8 gene—the most common molecular cause of severe hemophilia A, accounting for approximately 40%−50% of severe cases worldwide. Neuroimaging showed bilateral parietal bone rarefaction and scalp hematoma without acute intracranial hemorrhage.

Management involved fresh frozen plasma, plasma-derived and recombinant FVIII replacement, and phototherapy for hyperbilirubinemia. Following stabilization, the infant was transitioned to prophylactic emicizumab, which was well-tolerated; by 6 weeks of age, the cephalohematoma had nearly resolved with no further bleeding. This case underscores that neonatal-onset hemophilia, though uncommon, can present with potentially life-threatening hemorrhagic complications and must be considered in any neonate with unexplained anemia, prolonged coagulation times, or expanding extracranial hematoma. The successful use of emicizumab for secondary prophylaxis in a neonate represents an important clinical advance, as it avoids the inconvenience and risks associated with repeated intravenous infusions in infants.

## The mimicker: intracranial hemophilic pseudotumor

Among the most dramatic contributions to this Research Topic is the case report by Regmi et al. (Peking University Third Hospital and collaborating institutions, China), “*Case report: intracranial hemophilic pseudotumor mimicking an aggressive neoplasm: a rare skull-invasive presentation*,” published in *Frontiers in Surgery* in January 2026.

The authors describe a 36-year-old man with severe hemophilia A who presented with a 5-year history of progressively enlarging left frontoparietal scalp swelling and worsening right-sided hemiparesis. MRI demonstrated a large, heterogeneously enhancing extra-axial lesion measuring 10 cm × 10 cm × 5 cm with a “mushroom-like” extracranial extension, destruction of the left cranial vault, marked mass effect, and midline shift—initially interpreted radiologically as a possible meningioma. Under intensive intravenous FVIII replacement, the lesion was excised *en bloc* by neurosurgery; intraoperative findings revealed an encapsulated mass containing organizing clot and fibrous tissue. The skull defect was subsequently reconstructed in a staged procedure, and histopathology confirmed the diagnosis of hemophilic pseudotumor. Postoperatively, hemiparesis improved markedly.

Hemophilic pseudotumors are rare, chronic, expanding hemorrhagic masses that erode adjacent bone, typically arising in long bones and the pelvis. Intracranial presentations are exceedingly uncommon, and their ability to mimic neoplasms both clinically and radiologically makes diagnosis challenging. This case illustrates that hemophilic pseudotumor must remain in the differential diagnosis of any space-occupying intracranial lesion in a patient with hemophilia—and that surgical resection under meticulous hemostatic management can achieve excellent neurological outcomes even in advanced cases.

## The ultimate haemostatic challenge: open cardiac surgery in severe hemophilia A

Perhaps the most technically demanding of all surgical challenges in hemophilia is open cardiac surgery, in which heparin anticoagulation for cardiopulmonary bypass must be balanced against the profound coagulation deficit inherent to the condition. Ursu et al. (Children's Emergency Hospital “Louis Turcanu” Timi?oara, Iuliu Ha?ieganu University of Medicine and Pharmacy Cluj-Napoca, and collaborating Romanian institutions) address this challenge in their case report “*Successful concomitant surgical interventions for dilated ascending aorta and aortic valve insufficiency in a severe hemophilia A case*,” accepted in April 2026 in *Frontiers in Medicine*.

The authors present a 64-year-old man with severe hemophilia A (without inhibitors) who had developed cardiac failure secondary to severe aortic valve regurgitation and a dilated ascending aorta. The decision to proceed with concomitant surgical correction—aortic valve replacement with a biological prosthesis and reduction aortoplasty to a 35 mm diameter under cardiopulmonary bypass—was reached through a rigorous multidisciplinary team process in strict adherence to international hemophilia management guidelines.

The perioperative strategy required the simultaneous administration of FVIII concentrate (to correct the hemophilic baseline), heparin (for extracorporeal circulation), antifibrinolytics, and platelet management, all while navigating the additional physiological stressors of surgical trauma, cardioplegia, and hypothermia. Cardiopulmonary bypass was successfully weaned after 80 mins; the patient was managed in the intensive care unit for 2 days and discharged after 10 days. Postoperative anticoagulation was tailored as continuous FVIII prophylaxis combined with low-molecular-weight heparin for 3 months, followed by aspirin. The immediate and long-term outcomes were excellent, with no bleeding, thrombotic, or other complications recorded.

This case report is particularly valuable in demonstrating that, with meticulous multidisciplinary planning, laboratory-guided personalized hemostasis monitoring, and adherence to contemporary guidelines, open cardiac surgery can be performed safely even in patients with severe hemophilia A. The authors appropriately emphasize that continuous laboratory monitoring was the keystone of safe management, and that balancing conflicting hemostatic requirements—simultaneously preventing both hemorrhage and thrombosis—represents one of the most complex clinical problems in contemporary hematology.

## Beyond hemophilia: myelodysplastic syndromes and precision hematology

The final contribution brings a broadened hematological perspective to the Research Topic. The review by Zhang et al. (The 940th Hospital of Chinese PLA and Northwest Minzu University, Lanzhou, China), “*Hotspot gene mutations and treatment response in myelodysplastic syndromes (MDS): predictive biomarkers and targeted strategies*,” published in *Frontiers in Medicine* in January 2026, examines the genomic underpinnings of treatment response in another major clonal haematopoietic disorder.

Drawing on next-generation sequencing data, the authors systematically map the relationships between recurrent hotspot mutations—including those in ASXL1, DNMT3A, TET2, RUNX1, BCOR, EZH2, TP53, and IDH1/2—and the responsiveness of MDS patients to hypomethylating agents (HMAs), conventional chemotherapy, and targeted therapies. Key findings include: ASXL1 mutations predict HMA resistance, but the addition of venetoclax restores objective response rates to 87%; DNMT3A R882 mutations sensitize cells to decitabine via hemi-methylated enhancer trapping; TP53 multi-hit lesions, though carrying a dismal prognosis (median OS below 12 months), show remarkable responses to the p53-reactivating agent eprenetapopt combined with azacitidine (ORR 73%); and IDH1/2 inhibitors achieve durable remissions (ivosidenib ORR 83.3%, median OS 35.7 months).

While MDS is not a bleeding disorder *per se*, it shares critical pathobiological ground with the hemophilia field: both are conditions in which genomic precision medicine is fundamentally reshaping treatment algorithms, and both highlight the increasingly central role of molecular profiling in clinical decision-making. The inclusion of this review in the Research Topic reflects the thematic ambition to situate hemophilia advances within the broader context of precision hematology, reminding readers that genomic insights—whether in F8 mutation analysis, inhibitor risk stratification, or MDS mutation profiling—are now indispensable tools for the modern hematologist.

## Synthesis: converging themes and future directions

Taken together, the contributions to this Research Topic illuminate three converging themes that are likely to define hemophilia care in the coming decade.

**First, precision medicine is now inescapable in hemophilia**. Population pharmacokinetic modeling (Terasaka and McKeand), mutation-guided diagnosis (Peng et al.), and genotype-phenotype correlations (Dushimova et al.) all point toward an era in which treatment decisions—factor product choice, dosing interval, prophylaxis intensity—are individually tailored to each patient's molecular and pharmacological profile. This shift demands investment in laboratory infrastructure, genomic testing, and clinical expertise, particularly in resource-limited settings.

**Second, the therapeutic frontier continues to advance rapidly but unevenly**. Non-viral gene therapy (Kao et al.) and emicizumab for neonatal prophylaxis (Peng et al.) represent genuine innovations that may reshape the disease course for future patients. Yet as Dushimova et al. powerfully remind us, approximately 70% of patients worldwide today do not have access to the therapies that are already available. Bridging this access gap is as urgent a priority as developing the next generation of treatments.

**Third, rare and severe complications of hemophilia demand clinical vigilance and institutional expertise**. The cases of intracranial pseudotumor (Regmi et al.) and cardiac surgery (Ursu et al.) underscore that hemophilia can produce life-threatening emergencies that tax even the most experienced multidisciplinary teams. Both cases demonstrate that with meticulous hemostatic management, previously considered unsurvivable or inoperable scenarios can be successfully navigated. Disseminating such institutional experience through case reports and guidelines is essential for raising standards globally.

The editors express sincere gratitude to all contributing authors, reviewers, and editorial board members whose expertise and commitment made this Research Topic possible. We hope that these manuscripts stimulate further research, foster interdisciplinary collaboration, and ultimately contribute to a world in which every person with hemophilia—regardless of geography or socioeconomic circumstance—has access to safe, effective, and personalized therapy.

